# Using ‘Theories of Change’ and responsive feedback to design a digital service business for patent and proprietary medicine vendors in Nigeria

**DOI:** 10.12688/gatesopenres.13028.1

**Published:** 2019-06-13

**Authors:** Richard L. Wright, Abi Gleek, Nora Bergin, R. Algy Williams, Sohail Agha

**Affiliations:** 1 Chief Sustainability Office, Unilever PLC, Bebington, WIrral, CH63 3JW, UK; 2 Every1Mobile, Brighton, Sussex, BN1 4GW, UK; 3 Integrated Delivery, Bill & Melinda Gates Foundation, Seattle, WA, 98109, USA

**Keywords:** Theory of Change, Responsive feedback, Patent and Proprietary Medicine Vendors, Primary healthcare, Nigeria, digital

## Abstract

In a paper titled “Responsive feedback: Towards a new paradigm to enhance intervention effectiveness”, Viswanath *et al*. argue that dominant models of intervention design do not account for the complexity and unpredictability of implementation challenges.  Particularly in the behavioural sciences, intervention designs need to consider many factors that will be uncertain, or unknown, at the beginning of a new project.

This letter describes how we were able to respond to feedback during the design phase of a proof-of-concept project to create a digital service business for Nigerian patent and proprietary medicine vendors (PPMVs).  Our approach was to create an initial ‘Theory of Change’ (ToC) based on a similar project with Kenyan shopkeepers.  This ToC was revised following user feedback and a landscape analysis with key stakeholders.  The new ToC required us to access additional funding to create a ‘digital ordering’ facility for the PPMVs.  Digital ordering provides a mechanism whereby we can reduce the prevalence of counterfeit medicines, offer the PPMVs credit and group-buying facilities, and reduce supply chain costs through co-distribution with fast-moving consumer goods.

An important learning point was that while our focus was on designing a platform to meet users’ needs, changes in regulation meant that we spent considerably more time than anticipated meeting the needs of multiple stakeholders. However, the importance of ensuring stakeholders’ continued buy-in cannot be underestimated and has likely increased the sustainability of the project in the longer term.

As Viswanath *et al*. suggest, for responsive approaches to be widely adopted needs more flexibility than exists in current funding models and project plans.  Both funding bodies and grantees will need to be more responsive to feedback coming from the field.

## Introduction

Viswanath *et al*. (2019) note that current dominant models of intervention design do not account for the complexity and unpredictability of implementation challenges. In the behavioural sciences, intervention designs are affected by the enabling environment (political, regulatory, and structural factors) as well as social, cultural, and economic characteristics of the target beneficiaries. Given that many of these factors will be uncertain or unknown at the beginning of a new project, it would seem reasonable to allow time to define interventions before finalising budgets and timelines. This letter describes how we responded to feedback during the design phase of a proof of concept project to create a digital service business for Nigerian patent and proprietary medicine vendors (PPMVs). 

A Nigerian PPMV is defined as ‘a person without formal training in pharmacy who sells orthodox pharmaceutical products on a retail basis for profit’ ( Brieger *et al*., 2004). PPMVs constitute an important component of the private sector health system, particularly for poorer and more rural communities but are not well supported to provide quality health services ( Beyeler *et al*., 2015). However, with so many medically trained cadres operating as PPMVs, an opportunity exists to use them to improve and expand services for primary healthcare and family planning. The need to strengthen this vital health care delivery channel has been recognised by national and state-level government. We believe that a key component of this must be to establish a mechanism to support the PPMVs that both improves their service delivery while, at the same time, meeting their business and financial needs. This is the aim for NaijaCare.

The project described here was set up to deliver a proof of concept concerning the viability of NaijaCare, a for-profit digital service for PPMVs, which would allow them to improve their service delivery while growing their businesses. As such, part of the project involved identifying potential revenue streams for a digital service business which was also capable of delivering positive economic, social and health impacts for the PPMVs and their customers.

### UJoin

The inspiration for NaijaCare came from UJoin ^1^; a digital service for shopkeepers (known as ‘duka owners’) in Nairobi’s slums. UJoin provides duka owners with a digital community, which they access using their mobile phone. The community provides education and mentoring, credit to buy stock, and a ‘loyalty scheme’ through which discount vouchers are sent to their customers’ mobile phones. 

**Figure 1.**
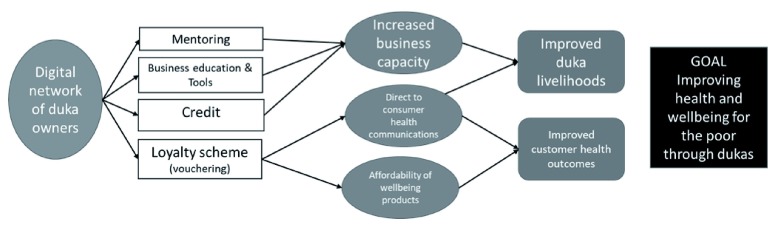
Theory of change for UJoin.

The ‘loyalty scheme’ is key to UJoin’s ToC as it allows us to create demand for some of the dukas’ stock in two ways (see Figure 1). First, we improve product affordability by sending discounts to customers’ phones. Second, the loyalty scheme provides a ‘direct-to-consumer’ communications channel. This is realised through UAfya, a digital community that provides young women with health education and peer-to-peer sharing on a broad range of issues. UAfya not only drives demand for some of the products stocked by dukas but also enhances the broader wellbeing of the young women. 

UJoin was set up on the premise that we could meet the needs, and drive behavioural change, for low-income households by leveraging the relationships between the duka owners and their customers. We felt that using this market-based approach could have benefits over more traditional direct contact approaches. First, the relationship between the duka owner and customer is long-term and based on trust and this could provide for a series of interventions that address multiple behavioural changes. Second, through the market we could create demand for consumables such as soap, menstrual health products, and nutritious foods; improve the livelihoods of duka owners; and create clear returns on investment for private sector partners investing in UJoin.

### NaijaCare

**Figure 2.**
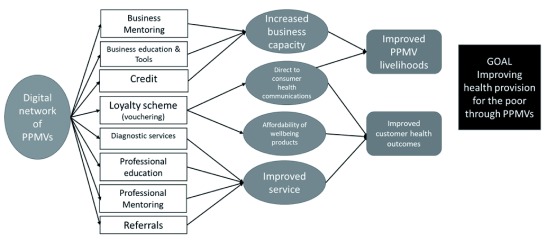
Theory of change for NaijaCare. PPMV, patent and proprietary medicine vendors.

We based the initial NaijaCare ToC on UJoin (see Figure 2). NaijaCare has the same business building blocks as UJoin but, in addition, we believed that there could be a route to impact by using the digital community to improve the PPMVs’ service delivery. There were three elements to this; (i) Education and mentoring to increase the quality of customer interactions; (ii) Support of the introduction of consumer diagnostics such as rapid diagnostic kits for malaria, which could identify consumer health problems and create further demand for PPMV products, and (iii) A referral mechanism, which in the first instance made it easy for the PPMVs to refer customers to providers of family planning services. The evidence is that current levels of PPMV referrals to clinics are low (see Beyeler *et al*., 2015 for a review).

## Designing NaijaCare

Initial design workshops with PPMVs revealed some similarities and some differences with duka owners. A key insight was that PPMVs saw themselves as business owners rather than health providers. So, like duka owners, their primary interest was in improving their businesses. There was a strong interest in credit for stock, need for stock control and business planning. While these insights did not affect our basic ToC, it became clear that the first release of NaijaCare needed to focus on business benefits rather than health provision.

In parallel, we conducted a landscape analysis, consulting with organisations such as the Pharmacist Council of Nigeria (PCN), the National Association of Patent and Proprietary Medicine Dealers (NAPPMED), and the Society for Family Health (SFH). What became clear from these consultations was that there were a range of ongoing developments, all of which would need to consider in our design. 

Key design changes resulted from reports that PPMVs are a major route for the supply of sub-standard and counterfeit medicines ( Health Communication Capacity Collaborative, 2016) and sell medicines that are not covered under their licence (e.g., Fajola *et al*., 2011–2012). Further, we discovered that imminent regulatory changes would create three tiers of PPMVs and require them to register with PCN. This context meant that the model built with duka owners was not enough to meet the requirements for PPMVs; a digital service that supported these businesses would need to mitigate the legal contraventions; facilitate any necessary registration; and target each PPMV with content that was appropriate to their tier. What had started as process of designing a digital platform that met users’ needs now needed to consider the needs of multiple stakeholders and gatekeepers. 

**Figure 3.**
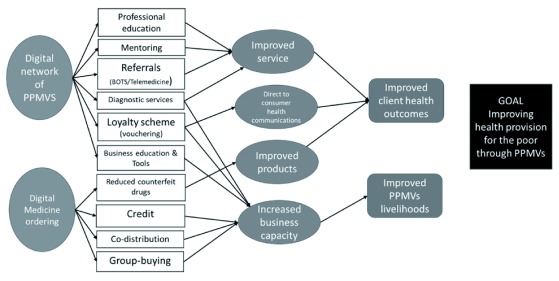
Revised theory of change for NaijaCare. PPMV, patent and proprietary medicine vendors.

Based on the landscape analysis and the business needs of the PPMVs, we felt we needed to alter our ToC (see Figure 3) to include a digital ordering facility that would enable PPMVs to order medicines from an assured provider. This required additional in-market relationships and technology development. Naturally, these additional activities led to a requirement to increase the budget envelope beyond that originally allocated. 

Digital medicine ordering allows us to create a secure supply chain of assured medicines, with PPMVs only able to access those medicines covered under their licence. Further, digital ordering can provide a digital trading history that may de-risk the provision of highly-valued credit, share distribution costs through adding further products to the basket (e.g. soaps, toothpastes, skin creams) and reduce the unit cost of stock for the PPMVs by enabling group-buying. 

It is our hope (to be tested) that that digital ordering will become a major reason for the PPMVs to engage with the platform and to conform with the relevant laws and regulations. It will reduce the potential friction in their lives and can be used to reassure their customers of the quality of their products. Finally, we hope that it could provide an affordable way for PPMVs to register with PCN through taking small payments as part of each transaction.

## Discussion and conclusions

The transfer of an existing ToC provided the starting hypotheses for NaijaCare. This was tested through qualitative and ethnographic research in our user-centred design process. Further, the appropriateness in the new context was explored with a new set of stakeholders. This led us to change our ToC and add the development of digital ordering in parallel with the creation of our ‘Minimal Viable Product’ (MVP) community. The community has now launched and is being refined based on user interactions and feedback. In addition to qualitative feedback we are now introducing quantitative measures; for instance, if the completion rates on a course are low then we revise the course. 

Our expectation is that, once our intervention has stabilised, we will test it more formally, providing more quantitative evidence for its effectiveness and our theory of change. Eventually, we may choose to perform a randomised controlled trial. However, we believe that it is important to spend considerable time in a more responsive mode – designing data gathering to enable decision making rather than as proofs of efficacy.

We would support the call by Viswanath *et al.* for a more flexible approach to behavioural interventions, whereby a theory of change is specified together with an associated level of uncertainty. If this can be accepted by all stakeholders (donors, governments, grantees), then projects can be designed in such a way to maintain flexibility in the early stages, including the use of responsive feedback mechanisms.

## Data availability

No data are associated with this article.
