# Supplementary material for: Using ‘Theories of Change’ and responsive feedback to design a digital service business for patent and proprietary medicine vendors in Nigeria
Source: Gates Open Res. 2019 Jun 13;3:1493. [Version 1] doi: 10.12688/gatesopenres.13028.1 (PMC6993493; doi:10.12688/gatesopenres.13028.1)
Supplement: Peer review history

# Peer review: Reviewer response for version 1

**DOI:** 10.21956/gatesopenres.14138.r27385

**Published:** 2020-01-24

**Authors:** Linda Fogarty

**Affiliations:** 1 International Youth Foundation, Baltimore, MD, USA

This open letter - “Using ‘Theories of Change’ and responsive feedback to design a digital service business for patent and proprietary medicine vendors in Nigeria” - provides good rationale for the letter, is accessible and well-referenced, and recommendations are clear. The letter is well-written and timely: the need for a more flexible approach to international health intervention design given the complexity and unpredictability of many global health challenges and contexts is even acknowledged by USAID through investments in Developmental Evaluation (through the Global Development Lab/Monitoring, Evaluation, Research and Learning Innovation Program). However, the letter does not spend time describing differing views and opinions. Though those differences may be obvious, it would strengthen the letter to add a sentence or two describing the current donor and development trends and expectations that make longer, more flexible intervention design periods difficult or even unacceptable. However, the letter makes a very clear and compelling case for such an approach. 

 I have a couple of questions about the note. First, affordability of well-being products was an outcome of the loyalty scheme in Kenya and in the first TOC for Nigeria. Why was it dropped for the final TOC? Though I realize the TOC is quite focused on the PPMVs, the goal of improved client health outcome seems directly supported by affordability. Second, and a very minor question, why the change from “consumer” to “client” to describe beneficiary of improved health? In this case, i.e., for this letter, it might be useful to harmonize these (just because this probably isn’t the right place to discuss such a detail) and so it doesn’t distract.

 Finally, I noticed two places where very minor edits might be helpful:

- In the Designing NaijaCare section, para 2, last sentence: There is a “we” missing. I think it should be: “…all of which we would…”.
- Last paragraph on page 4, first sentence: “It is our hope…” there are two “that” words together. I think only one is needed.

I confirm that I have read this submission and believe that I have an appropriate level of expertise to confirm that it is of an acceptable scientific standard.

# Peer review: Reviewer response for version 1

**DOI:** 10.21956/gatesopenres.14138.r27386

**Published:** 2019-06-17

**Authors:** W. Douglas Evans

**Affiliations:** 1 Milken Institute School of Public Health, George Washington University, Washington, DC, USA

This is an interesting and valuable paper on the use of theories of change and responsive feedback to improve a digital business model for patent and proprietary medicine vendors in Nigeria. The impact of this project is potentially very large and the study is well conceived and the paper well written and clear. I have just a few minor suggestions for revisions:

1. It is worth noting that the Viswanath *et al.*, 2019 paper and the responsive feedback model in general is part of a much larger literature on evidence<-->practice feedback loops. The Precede-Proceed model, RE-AIM, and related theories which are part of this tradition, have been studied extensively, and should be noted as background. Responsive Feedback is an extension and outgrowth of these well established models.
2. It would be valuable to readers if the article said more about the data that were gathered as the project in Nigeria developed and how those data were analyzed and interpreted. What specific findings led to changes in the ToC reported here? More specifics would be helpful.
3. How will we know if the program in Nigeria is a long-term success? The authors could provide more discussion on potential future research, questions to be answered, and challenges likely to be faced.
4. The article overall is a bit short on citations to other research related to evidence<-->practice feedback loops, which relates back to point 1.
5. This project clearly applies a marketing framework and approach, yet marketing is never mentioned nor are there any citations to relevant literature on marketing theory and methods.

I confirm that I have read this submission and believe that I have an appropriate level of expertise to confirm that it is of an acceptable scientific standard.
